# A novel *ATP2C1* mutation (c.1840-1G>A) in a sporadic case of isolated perianal Hailey-Hailey disease with human papillomavirus type 58 infection^☆^

**DOI:** 10.1016/j.abd.2022.12.011

**Published:** 2024-04-17

**Authors:** Yao Zhu, Yi-Ming Fan, Yan-Xia Cai, Yong-Hua Chen, Fang Qiu

**Affiliations:** Department of Dermatology, Affiliated Hospital of Guangdong Medical University, Zhanjiang, Guangdong, China

Dear Editor,

Hailey-Hailey Disease (HHD), or familial benign chronic pemphigus (OMIM:169600), is a rare autosomal-dominant blistering disease characterized by recurrent blisters, erosions, and macerated plaques mainly involving the intertriginous regions. It is caused by *ATP2C1* mutation, encoding secretory pathway Ca^2+^/Mn^2+^-ATPase 1 (SPCA1).^1^, ^2^ Updated on May 16, 2022, 264 public variants have been documented in *ATP2C1* LOVD database (https://databases.lovd.nl/shared/genes/ATP2C1). We report a sporadic case of isolated perianal Hailey-Hailey disease caused by a novel splicing mutation of *ATP2C1* with Human Papillomavirus (HPV) 58 infection.

A 21-year-old Chinese man presented with a three-year history of perianal itching, erythema, and macerated papuloplaques (Fig. 1A) in September 2021. He had a ten-year history of chronic diarrhea, no family history of similar dermatosis, and no history of sexual activity. Serologies for syphilis, HIV and herpes simplex virus were negative. Colonoscopy findings were unremarkable. Based on positive dot blot hybridization of HPV58 which was performed by using a smear from the lesion and a suspiciously positive acetic acid white test, he was tentatively diagnosed with genital warts and treated with electrofulguration and three sessions of 5-aminolevulinic acid Photodynamic Therapy (PDT). The lesions were alleviated but relapsed one month post-treatment and HPV reexamination (using smear from the lesion) was negative. Lesional biopsy showed epidermal hyperkeratosis; focal parakeratosis and dyskeratosis without koilocytosis; suprabasal acantholysis with “dilapidated brick-wall” appearance; and mild perivascular lymphohistiocytic infiltrate in the upper dermis (Fig. 2A). No comparable lesions were observed on other body regions. A final diagnosis of HHD with HPV58 infection was made. The lesions responded poorly to oral methylprednisolone, cyclosporine, methotrexate, topical corticosteroids, antibiotics, and tacrolimus for three months. Sanger sequencing of the patient’s peripheral blood revealed a novel heterozygous splicing mutation c.1840-1G>A (p.?) in intron 19 of *ATP2C1* (NM_014382.4), and a wild-type sequence in his parents (Fig. 3). Immunohistochemical staining of SPCA1 polyclonal antibody (PA5-109430; Invitrogen, Carlsbad, CA, USA) revealed that epidermal expression was lower in the HHD lesion than in normal perianal skin of the other normal patient (Fig. 2B‒C). The patient underwent ultra pulsed nonablative CO2 laser therapy, achieving mild lesional improvement with markedly improved pruritus (Fig. 1B).

**Figure 1:**
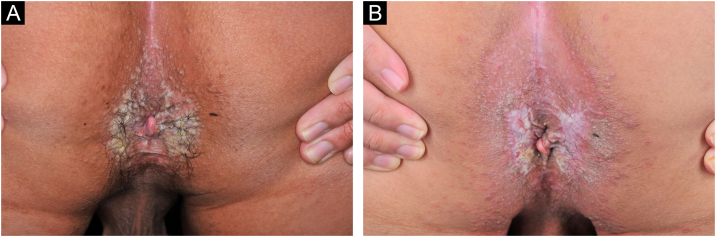
Clinical observation. (A) Perianal erythema and macerated papuloplaques. (B) Mild lesional improvement 6-months following CO2 laser.

**Figure 2:**
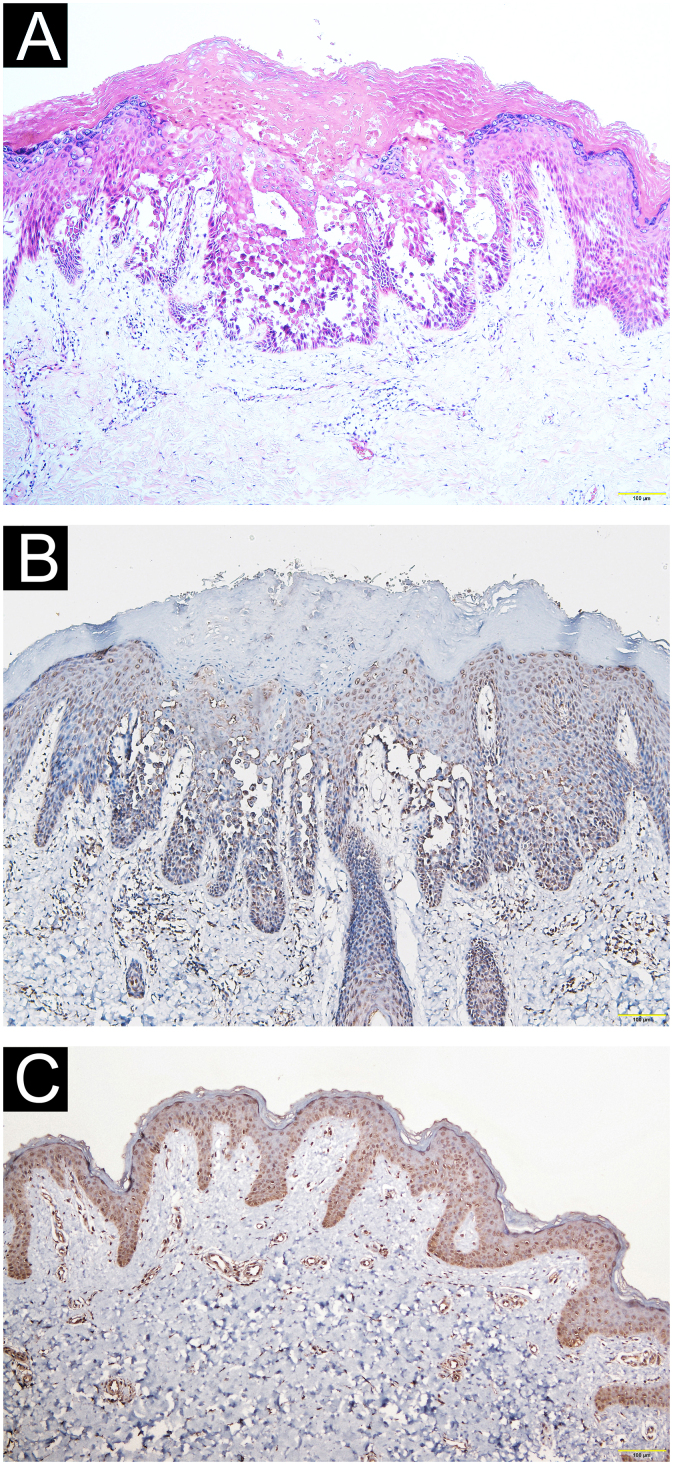
Pathological and immunohistochemical observation. (A) Epidermal hyperkeratosis, focal parakeratosis and dyskeratosis without koilocytosis, suprabasal acantholysis with “dilapidated brick-wall” appearance, and mild perivascular lymphohistiocytic infiltrate in the upper dermis (Hematoxylin & eosin, ×100). (B‒C) Immunohistochemical staining revealing reduced epidermal hSPCA1 expression in the Hailey-Hailey disease lesion (B) compared with normal control skin (C) (×100).

**Figure 3:**
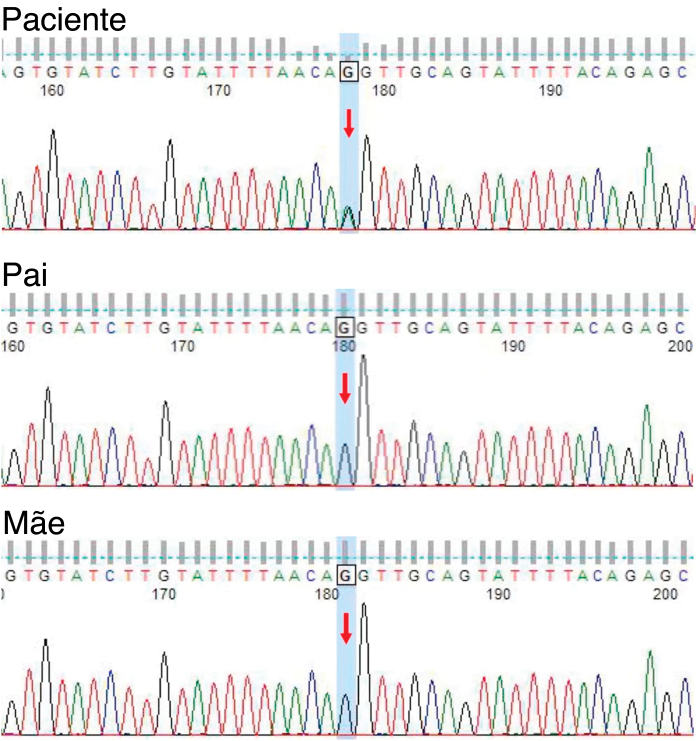
Genetic detection. Sanger sequencing of peripheral blood revealing a novel heterozygous splicing mutation c.1840-1G>A in intron 19 of *ATP2C1* in the patient, and a wild-type sequence in his parents.

This patient harbored a novel heterozygous *ATP2C1* mutation c.1840-1G>A at the acceptor splice site of intron 19. Mutation Taster analysis predicted that this variant may be a pathogenic splicing mutation and cause SPCA1 dysfunction by affecting magnesium binding sites.^1^, ^3^ Consistent with a previous report,^2^ SPCA1 immunoreactivity was reduced in the HHD lesion compared to control skin. *ATP2C1* mutations lead to defective calcium homeostasis and absent interkeratinocyte adhesion.^4^

Patients with HHD are susceptible to infections owing to skin barrier damage, but few reports exist of HHD concomitant with HPV6, 16 and 39 infections.^4^ Since typical cauliflower-like papules and genital involvement were absent, and PDT resulted in negative HPV58 and koilocytosis, it is unclear whether the HPV58 positivity represented transient colonization or subclinical infection in this case. Nevertheless, HPV infection could complicate the course and prognosis of HHD.^4^

While there are no treatment guidelines for HHD, ablative surgery including dermabrasion, CO2 and Er:YAG laser therapy, and argon plasma coagulation may be effective.^5^ However, the clinical efficacy of nonablative CO2 laser therapy and PDT was unsatisfactory in this case.

## Financial support

None declared.

## Authors’ contributions

Yao Zhu: Final approval of the final version of the manuscript; drafting and editing of the manuscript.

Yi-Ming Fan: Final approval of the final version of the manuscript; writing of the manuscript or critical review of important intellectual content; the study concept and design.

Yan-Xia Cai: Final approval of the final version of the manuscript; effective participation in the research guidance; intellectual participation in the propaedeutic and/or therapeutic conduct of the studied cases.

Yong-Hua Chen: Final approval of the final version of the manuscript; effective participation in the research guidance.

Fang Qiu: Final approval of the final version of the manuscript; critical review of the literature.

## Conflicts of interest

None declared.
